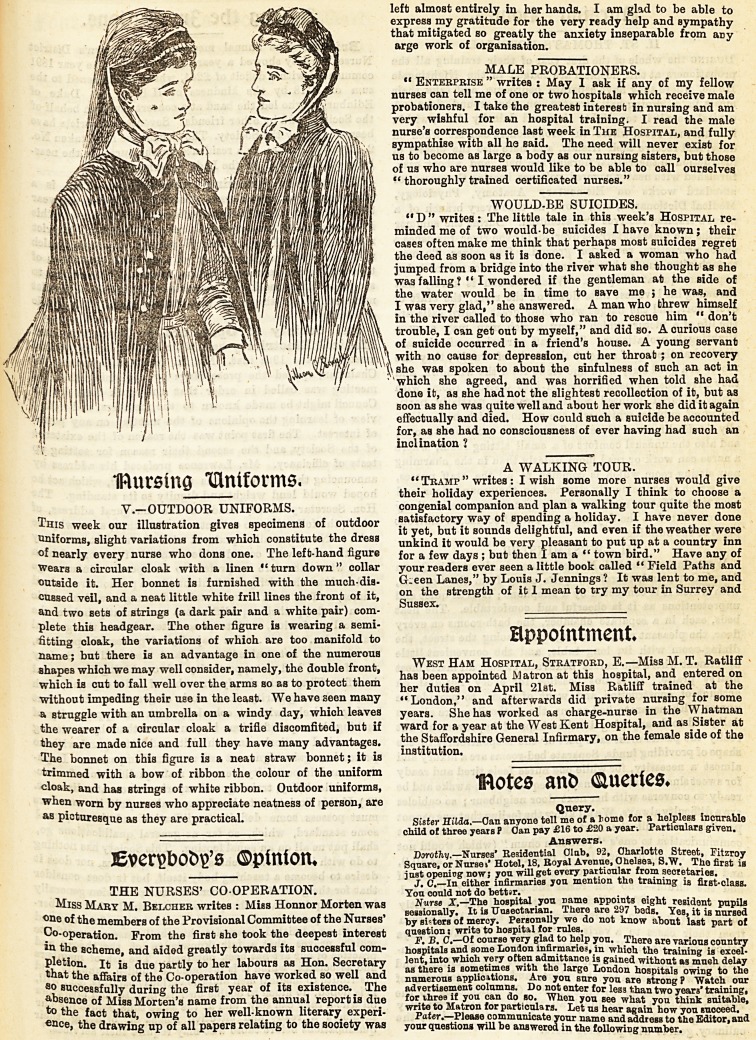# The Hospital Nursing Supplement

**Published:** 1892-04-30

**Authors:** 


"The Hospital" Nursing Mirror.

Being the Extra Nursing Supplement of "The Hospital" Newspaper.

Contributions for this Supplement should be addressed to the Editor, The Hospital, 140, Strand, London, W.C., and should have the word "Nursing" plainly written in left-hand top corner of the envelope.

### En Passant.

The Co-operative System.—Miss Harbord, the Lady Superintendent of the Nurses' Institute at Margate is taking a certain number of nurses who receive their own fees, minus a commission of 1s. 6d. in the guinea. There is an entrance fee of a guinea, and 18s. per week is payable in advance for board and lodging when the nurse is not at a case. Medical, surgical, and monthly nurses are on the books. We are shortly to hear of more co-operation at Brighton.

Observant Womankind.—Dr. Schofield, in his excellent paper which he read at the Congress of Hygiene last year, paid a tribute to the gentler sex which we cannot refrain from quoting, at the risk of wounding the susceptibilities of the sterner sex: "As a rule, a man knows very little about a house beyond the rooms he occupies. It is the mistress who inspects the lower regions, who knows where the cistern is, who sees the dustman call, and who scents incipient sewer gas. How important, then, nay, how absolutely essential it is, that the young girls of our country, ere they marry, should be carefully trained in the knowledge of what pure air and what pure water mean, and the causes and varieties of domestic poisons."

Nursing at the Royal Free.—The report of the sixty-f urth year of this hospital is very complete and interesting. Dr. Sainsbury and Mr. Barrow have been the lecturers on medical and surgical nursing, and the annual examination of probationers shows that it has not been labour in vain. Amanda Jones won the gold medal for the highest number of marks. The private nurses of this hospital seem to have been in great demand, the applications for their attendance having generally been in excess of the number available. The year has seen Miss Barton leave her post to be married, and the place has been filled by Miss Henrietta Wedgwood. The Ladies' Visiting Committee is so far working happily, and their efforts seem to have been of assistance to the weekly Board.

A Pleasing Comparison.—Last week's *British Medical Journal* contains a letter from a medical man which is an interesting contrast to the opinion of another doctor anent nurses and their work. How lucky for us all that opinions differ. One gentleman finds bad nursing very far from being exceptional; the other finds that, in spite of much hardship and discouragement incident to their calling, that nurses may be truthfully spoken of as a very superior body of women. It is very good for us to hear the opinions of medical men, adverse and otherwise; there are always some points which make useful hints to be gathered from these observations. We are none of us so conceited that we do not know that the standard of nursing excellence is not all we want it to be, nor are our ranks free from those who give "bad nursing." The antidote to these difficulties is excellence of training. Each nurse must be individual in her efforts to bring forward the best result. Each year brings a higher standard for us to attain, and so the training must increase proportionately. Just a few words on the "hardship" we quoted. Many of us know the lines: "I say the happiest people, the fondest of their occupation, the most thankful for their lives, are, in my opinion, those engaged in sick nursing. It is a mere abuse of words to represent the life, as is done by some, as a sacrifice and martyrdom."

Lerwick.—A nurse seems badly wanted for this place, judging by the correspondence in the *Shetland Times*. One writer suggests that the Lerwick Sick Aid Society might include the question of a nurse in their scheme. So we should think. The idea of a town without a trained nurse makes it behind the times, wherever that town may happen to be.

The Royal British Nurses' Association.—It has been often stated that the names of nurses and others, who have ceased to pay their subscriptions to the R.B.N.A., and who have so retired from membership, find that their names still appear in the published lists and reports. Everyone so circumstanced is invited to send her name, with a brief statement of the fac s, to the Treasurer, St. Thomas's Hospital, Albert Embankment, London, E.C.

Bygone Uniforms.—The authorities at St. Thomas's Hospital did wisely when they cancelled the old rule which obliged the Nightingale probationers to go out on Sundays in a grey dress and shawl, with a white straw bonnet, trimmed severely with straight grey ribbon, at a period when unfamiliarity made all nursing uniforms conspicuous in public places, and this dress, suitable and becoming as it may have been, must have rendered church-going a curious ordeal for the modest aspirant in the training school of those early days.

Nurses and Religion.—Professor Virchow has been engaged in lamenting the intrusion of religious arrogance into hospital work. The Professor finds that creeds assert supremacy in the healing of the sick; the spirit of humanity ought, says he, to be alone at the sick bedside, and the conflict of creed ought to be kept away. Luckily for us, we suffer very little from anything of that sort; religious bitterness, though it seems far from being blotted out altogether, has had its sharp edges considerably blunted, and the platform of common kindness is, we can thankfully say, made a meeting ground for every form of creed or dogma. But it is well to remember that, although we in England can deny that "creeds claim supremacy" here, Virchow is right in saying that the *spirit* of humanity alone ought to be at the bedside of the sick. "To live and do the right," this is the practical result of all religion, before which dogmatic disputes fade in their uselessness. The *true* spirit of humanity is divine.

Short Items.—The Original Company of Amateurs will give "The Golden Apple" at the New Pier Pavilion at St. Leonards on Monday, May 2nd, the funds to go to help the Hertfordshire Children's Convalescent Home, St. Leonards.—The Duchess of Rutland says that the number of cases of entire reformation from drink increases steadily. —Lady Zetland visited the Rotunda Hospital last week and complimented the authorities on the marked improvement made since her last visit.—Miss C. O'Conor Eccles is contributing a series of articles on the London hospitals to the *Health Record*, the Irish journal of sanitary science.—A bazaar in aid of the parish of St. Gabriel, Poplar, is to be held at the Bow and Bromley Institute on May 11th, 12th, and 13th. The hall is to be arranged as an "Olde Englishe Village," under the direction of Sir James Linton. Fancy dress concerts and organ recitals are among the entertainments. The single entrance-fee to the bazaar gives admittance without extra charge.

### Ventilation, Disinfection, and Diet.

By P. Caldwell Smith, M.D.

### III.—PURE AIR AND NATURAL VENTILATION.

The air which is allowed to come into inhabited rooms must be taken from a pure source; there should be no possibility of it being contaminated before entering the room. In hospitals, for example, if the air is drawn through tubes or below the floors, these should be kept clean and free from dust. The air must also in hospitals be of proper temperature, and means must be adopted, if the ventilation is to be at all satisfactory, to warm or cool the air as is required. A very important thing to remember is that you must not only have openings for the escape of foul air (outlets) but you must also have openings for the admission of fresh air (inlets). This is often omitted by many architects. I know of several public buildings where outlet after outlet has been added to provide, as is thought, for the escape of foul air, without providing inlets for fresh air, and the consequence is that draughts are produced from doors, crevices in windows, &c., and some of the so-called outlets are converted into inlets.

Ventilation is divided into (1) natural ventilation and (2) artificial ventilation. In natural ventilation there are three main forces at work: (1) Diffusion of gases, (2) force of the wind, and (3) expansion of the air by heat. The first, diffusion of gases, may be practically left out of account, as it is comparatively small, and is totally insufficient for ventilating purposes.

The meaning of the term diffusion of gases may be explained thus. Take two specimens of air at different temperatures, then the weight of a certain amount of the one differs from the weight of the other, according to its temperature. If then these two specimens be placed in a jar with porous material between them, in a short time they will mix. Now this occurs through brick or stone walls, but as I have stated, only to a very small amount.

We have, however, in the second force at work in natural ventilation, viz., the force of the wind, a very powerful ventilating agent. If a room contains windows at each side and these are opened and the wind allowed to blow through, you have what is technically called perflation, and you can easily see that the air of a room can then be changed very rapidly. There are, however, several objections to the use of the wind in this way. In the first place there may be no or very little wind, the air may be stagnant, and is called so when the velocity of the wind does not exceed one or one and a-half miles in an hour. This, however, does not very frequently occur in this country, the average annual movement being stated to be 6 to 12 miles per hour. Another objection is that the movement of the air is very uncertain, and it is almost impossible to regulate it. Of course, it produces very great draughts, and perflation should, therefore, only be used when the room is unoccupied. The wind, however, is often used as a ventilating agent in another way. When it blows over a chimney, it causes an upward current in the chimney. Many of the ventilators which you see placed on the ridges of churches, schools, hospitals, &c., depend on the action of the wind, an upward current being produced in the tube by the wind blowing across the ventilator, and then the foul air is extracted from the building. Perflation is used largely in ships, the wind blowing down the ventilators to the lower parts of the ship.

The third force at work in natural ventilation is expansion of gases of heat. The air of a room heated by respiration or by artificial means, such as open fire, hot-water pipes, gas, &c., expands and ascends to the ceiling. If there is any outlet there it will escape, but if not it will descend again at the coolest part of the room, as in front of the window. The air remaining at the roof or ceiling is in consequence very much lighter than an equal bulk of cold air outside, either in the open air or in passages or corridors. Consequently the cold air rushes in by special inlets, if these are provided, or if no such means exist, by any chinks or crevices. This goes on until the air is of the same temperature outside and inside, but the air inside is constantly being heated, and so the process goes on, and a constant current is kept up. Open fires when a fire is burning act pretty much in the same way, but they do not ventilate sufficiently, as the air above the level of the throat of the chimney, or rather above the mantelpiece, has a tendency to stagnate unless special means are provided for the escape of foul air higher up, nearer the ceiling. This is now often done by the introduction, about 18 inches or two feet from the ceiling, of an Arnott's Ventilator with Myle's mica flap valve. The hot air rushes through this into the chimney and is carried off. One objection to this is that these mica valves are rather disturbing, and especially so to a sick person when the wind is high, while another is that they are sometimes apt to get out of order, and as a consequence, smoke is forced into the room. Of course, a very much better arrangement is that these outlets should lead into a special flue heated by some means; but very few even of the most modern houses possess such flues. It may be said, however, that even chimneys without fires burning may act as ventilators by the wind blowing over the top of the chimney and causing up-draughts, but they may, and often are, converted into inlets under the following circumstances: (1) If a chimney is too low and commanded by higher buildings, the wind flows over them and down the chimney. (2) If two fireplaces are in one room, the one chimney may draw air down the other, and the strongest fire will cause a draught down the chimney with the weakest fire. (3) If when the fire is lighted there is not sufficient air entering the room, a down draught is created. The remedy, of course, for it is opening the door or window, thus getting a plentiful supply of air. (4) Occasionally fires lighted in grates in top rooms may draw air down the chimneys opening into the lower rooms.

This cause of movement of the air, viz., its expansion by heat, is continually in operation, as the temperature of the air is always changing. It must be remembered, however, that it can only act when there is a difference of temperature between the outside air and that in rooms, and consequently its action is greatest in winter, when the rooms are artificially heated. It may be necessary to determine the direction and velocity of the air currents. To determine the direction, a piece of brown paper may be burned, and the way the smoke is carried watched. It may be taken for granted that when there is a fire in a room all the air currents from different parts of the room—below door, window crevices, &c.—go towards the chimney, and if no other outlet exist, by measuring, with an instrument called an anemometer, the discharge from this chimney, the number of cubic feet of air entering the room may be easily obtained; this instrument is used daily in coal mines for determining the amount of ventilation of them. The inlets for the admission of fresh air should be placed as low as possible, but draughts should, of course, be avoided, and this may be done in various ways. In the first place, the inlet may be placed six or eight feet from the floor, and this requires to be the height if no artificial means are used for warming the air. There is a special form of apparatus used, called Tobin's tubes, which are now becoming very common, not only in halls, churches, and schools, but also in dwelling-houses. They are simple and very effective, but the great fault in all public edifices is that there are not nearly enough of them. They should not be very large individually, as the current may be too strong, but if introduced at suitable places are a great help in purifying the air. The air is taken directly from the outside at floor level. A grating being placed in the wall, it is let upwards, and a cone of gauze is placed at the mouth of tube, and in some cases a layer of light cotton wool, to catch dust particles. Of course, a much more satisfactory arrangement is to have the air heated. If the air is heated it may be introduced from three to four feet from the floor, and this is the method which ought to be adopted in all hospitals, in which no mechanical system of ventilation is used. The air should be led over coils of hot-water pipes placed between the windows.

### The Nursing of the Insane.

By a Medical Officer.

### I.—WHY TRAINING IS NECESSARY.

Is any special training needed in order to make a woman an efficient nurse in the medical wards of a hospital? This is an absurd question to ask now-a-days, and he who makes the enquiry would be answered with a smile of pitying derision. If, however, he seek to find out whether any instruction is needed by her who takes charge of those suffering from diseases of the brain, which call forth the highest qualities a nurse can possess, he will find that in practice it is considered that for these affections the light of nature is enough. Strange as it may sound yet it is true that, up to quite a recent date, it was thought that no training nor instruction was necessary for those who took charge of the insane. Whatever knowledge of her duties the nurse possessed she picked up for herself, and as a rule, went through her daily routine without a spark of intelligent interest in her work. I have known persons who have been in charge of wards for years, and yet the idea that those under their care were suffering from a bodily disease never once crossed their minds.

For affections of all organs but one the public now demand skilled nursing; in the very cases in which it is not obtained is it of most avail. Good nursing in lunacy is all-essential. The idea is still prevalent that insanity means a disease of something apart from the body; that for this affliction a prison is necessary rather than a hospital; and that those who tend the sufferers are less nurses for the sick than "keepers" in a house of detention. We have advanced far in our efforts to make the treatment of the insane more humane and more in keeping with the greater light now thrown on the disease. Great success has attended the efforts. Methods of kindness and mercy have taken the place of the cruelty and coercion which stained every page of its history. No longer can the scenes described by Conolly, the descriptions of which cannot be read without a shudder, be seen in our land. The light of heaven has been let into the dungeons; shackles, chains, and other instruments of torture have been banished; brutal malefactors accompanied by savage dogs no longer enforce their own more savage decrees. That period when the shadow of death hung over the lunatic has passed never to return. The horrors then perpetrated were unjustifiable on the grounds of morality and they were indefensible on the grounds of expediency.

Thus far we have gone, but another step is still to be taken. The next advance in our treatment of lunacy must be made in the education of our nursing staffs. As we have said, the status of the asylum attendant is less that of a nurse than that of a prison warder; the key is more symbolic of her duties than the feeding cup. It is not difficult to see how this now false view of the question sprang up, became firmly rooted, and is now fostered. Insanity in the past was a crime to be punished with manacles and flogging; to-day the idea still survives, but the asylum is now a house of detention rather than one of correction. We want it to be looked upon as a hospital, but that will never be until the public learn to look upon those employed in it as having a higher function than that of keeping the lunatic locked up from the world. So firmly has this idea become fixed, that it is a great hindrance to the early treatment of the insane.

*(To be continued.)*

### "THE POINT OF VIEW."

How strangely does a building vary in its appearance when seen from different points of view. As one travels by railway, for instance, a cathedral looms in the distance; at first it appears on one side of the road, then on the other as we draw nearer, and closer still it becomes alternately a long building, a short one, a number of mean straggling details or a grand massive pile. In like manner a women may look beautiful or plain accordingly as her profile or full face is presented to us. Mentally and morally, too, our views equally differ. The man or woman of from thirty to forty years, is voted "old" by the juveniles, whose ideas will modify as they themselves grow nearer that age; while to those who are dropping or have dropped into the "sere and yellow leaf" it is the prime and vigour of life. Again, an economical person will be considered a skinflint by the spendthrift and a prodigal by the miser, each view being quite the reverse of the proper one.

If there are such diversities in men's minds, how much happiness or misery depends, then, on the way we look at our own lives. There is a bright side and a dull side to them we all know, but by putting them into the right focus we can always keep the best one foremost. Any one who starts with the notion that life is to be idled through in a round of self-indulgence, who calls everything which opposes it a bore, and all work a grind, is in a fair way to arrive at the conclusion that life is not worth living. The bright and healthy soul, who works with a will, loves to help its fellow-creatures and keeps a sympathetic eye on everything, considers this a very excellent world indeed.

And which is right and which is wrong? When illness falls on us we shall soon find out the trials which bring us to perfection will prove which is man's view and which comes from God; and we must try to see with Him. He knows that we are not perfect, that sometimes our tempers last long, sometimes they are very short, sometimes we are good, sometimes bad, but He will help us to make of our hearts a beautiful and consistent whole even if outwardly to our neighbours the irregularities still appear.

The Christ who made allowance for the weakness and infirmities of men will teach us how much good there is in our friends and nurses, so that we may forgive them should they seem impatient with our requirements. Our aches and pains, so hard to bear, can be looked at in the light of Jesus' love, and they will turn into the flowers and blossoms which make our lives day by day more resemble His. Let us pray then to our heavenly Father, and ask that He will so form and fashion us that we may appear to all men a beautiful building standing four square to His glory, while we see others from such a point of view that their faults and failings will fade from our minds, and we shall dwell only on those good qualities which come direct from the great Architect's hands.

### Nurses for the English in India.

There seems to be some difference of opinion as to the practicability of the proposals made by Mrs. Edith E. Cuthell in the letter from her which we published last week. Surgeon-Colonel J. Butler Hamilton, M.D., P.M.O. W. Department, writes from Devonport to the *Times*, under date of the 22nd inst., as follows :—

There can be no doubt whatever of the great want of trained nurses in India, especially for the civil population living "up country"; but the question of supplying this want is not at all so easy as your correspondent seems to fancy. I may say that for the past twenty-seven years I have spent the greater part of my service in India, and long felt the want of a trained nursing staff in our military hospitals. This feeling was intensified on joining my last appointment—the charge of the Station Hospital at Lucknow in the spring of 1886, as, shortly after I took over the duties, I had no fewer than fifty severe cases of typhoid fever under treatment at one time, to say nothing of the numerous other bad cases of illness—such as abscess of the liver, pneumonia, dysentery, and the other ills of an Indian life. Medical officers I had in plenty, also a most excellent staff of warrant officers (apothecaries), but for nurses I had absolutely no one but the rough, hard-handed comrades of the sick. I ask any one to consider what this meant—fifty cases of typhoid fever, with perhaps some fifty other cases requiring good nursing, the thermometer standing 90 deg. in the wards and 115 deg. in the shade, and not one single nurse to attend on the sick. I was so impressed with the absolute necessity for trained nurses that I wrote and published a pamphlet on "Nursing in our Indian Military Hospitals," which was, by kind permission, dedicated to Lady Dufferin. This, I am glad to say, drew attention to the facts of the case. At the same time her Excellency Lady Roberts instituted her homes for nurses in the hills, and, in anticipation of the action of the Indian Government, raised funds and got out a number of excellent lady nurses, who were distributed among the military stations, and did good work in our hospitals till the arrival of the Government nurses. Now all is changed. The Government of India has a staff of most excellent and skilled lady nurses for duty in our military hospitals, and these can be sent by the P. M. O. of a district wherever required. During my five and a-half years' tenure of the charge of the Lucknow hospital we had ust 600 cases of typhoid fever to deal with, including officers, men, women, and children; this, be it remembered, in a garrison averaging 2,500 European soldiers. Compare this with the outbreaks (!) so-called in Dublin, where in a garrison of some 6,000 men twenty-five or thirty cases of enteric fever entailed an expenditure of thousands of pounds on the Royal Barracks, on the recommendation of sanitary commissions, *et hoc genus omne.* The people of England have little idea of the amount of sickness that occurs among the young officers and soldiers serving in India, and the medical staff of the army can point with pride to the fact that the mortality is, considering the circumstances, wonderfully low. During the five and a-half years I was at Lucknow we lost nearly seventy officers and men from enteric fever alone—a really small proportion when we think of the number of severe cases and the climate we had to contend against. I can, however, state with confidence that, since the arrival of our military nurses, medical officers have been able to treat their patients with much greater confidence, and that the results have been most satisfactory.

Who that saw them can ever forget the labours of Miss James, the Lady Superintendent, and her staff of Sisters during the outbreak of enteric last year? And I feel convinced that many valuable lives were thereby saved. I have entered into this digression in order that the public may know and understand the real necessity that exists for trained nurses in India. The question is how to give practical effect to Miss Cuthell's suggestion to supply the need. Miss Cuthell takes rather a sanguine view of the case when she writes: "Once in India a qualified nurse would make a very comfortable living; the pay is excellent, the demand large." Now, here I must differ from Miss Cuthell. There is no doubt that the "demand is large," but I regret to say the pay is not excellent, nor could a qualified nurse count on making an excellent livelihood—at all events, not unless she were one of a sisterhood, with a fixed home to return to during the intervals of employment. Rs. 150 (about £10 at the present rate of exchange) per month, with "travelling expenses" and "keep" while employed, is all the best of nurses could expect or ask for. It must be remembered that those most likely to require nursing are young civil or military officers, planters, clerks, &c., lately landed in the country. The pay of a young civilian is about Rs. 400, that of a second lieutenant Rs. 200 a month, and of the other classes less, and out of these sums all the expenses of living, rent, servants, keep of horses, &c., have to be met.

Admitting that a nurse gets Rs.150 a month and "all found," she must have a local habitation to which to return during the intervals of employment; and to those who know India and its expenses it will be evident that Rs.150 a month would be quite insufficient. Then there are clothes to be paid for, trips to the hills for a change, absolutely necessary, and all the various expenses of an Indian life. The Government of India, which never, so far as my experience goes, treats its medical servants with an excess of liberality, gives a *minimum* pay of Rs.175 per mensem to the junior lady nurses, with house rent, furniture, and many other advantages, and, as they live together, their expenses are, of course, comparatively low. I must not be understood as in any way desiring to throw cold water on Miss Cuthell's proposal, but I think it right that the "pros and cons." of the subject should be duly considered.

In the present condition of Indian finance, with the rupee fast falling to a shilling, and a party of loud-voiced "geese," as Sir Lepel Griffin aptly called them, cackling for the abolition of the monopoly in the growth and sale of opium, which would entail a loss of five millions sterling, no help can be looked for from the State. Lady Dufferin was able to raise an enormous sum of money to provide medical aid for the women of India; will no equally philanthropic lady take up the case of the young English men and women who perish yearly for want of proper nursing?

The only way, in my opinion, to provide "nurses for the English in India" is to raise a national subscription, and establish nurses' homes in central places, each under charge of a Matron, and from which nurses could be obtained upon application. In the Bengal Presidency, Lucknow, Amballa, and Rawal Pindi would be suitable places, with a "home in the hills" for the nurses to go to for a change. In Madras Bangalore and in Bombay Poonah would be suitable centres, with another at Jubbulpore for central India. To carry out such a scheme, or, indeed, any scheme likely to succeed, would require a very considerable capital, and I am convinced that it would only lead to disaster if nurses went out to India on their own account in the hope of obtaining a living. I am aware that there are one or two who have done so, but they have relations out there, with whom they live during the intervals of employment. I can only say in conclusion that my experience and assistance are at the disposal of any one who will take the matter up on the lines I indicate.

### Presentation.

Miss Annie Jays has resigned the post of Nurse-Matron of the Great Western Railway Hospital, Swindon, to resume that of charge-nurse at the Scarborough Sea-Bathing Infirmary, a post which she has previously held for three years. Before leaving Swindon the medical staff presented Miss Jays with a very handsome travelling bag, with silver fittings, as a mark of their appreciation of the excellent work she had done during the time she was at Swindon.

### Nursing Uniforms.

### V.—OUTDOOR UNIFORMS.

THIS week our illustration gives specimens of outdoor uniforms, slight variations from which constitute the dress of nearly every nurse who dons one. The left-hand figure wears a circular cloak with a linen "turn down" collar outside it. Her bonnet is furnished with the much-discussed veil, and a neat little white frill lines the front of it, and two sets of strings (a dark pair and a white pair) complete this headgear. The other figure is wearing a semi-fitting cloak, the variations of which are too manifold to name; but there is an advantage in one of the numerous shapes which we may well consider, namely, the double front, which is cut to fall well over the arms so as to protect them without impeding their use in the least. We have seen many a struggle with an umbrella on a windy day, which leaves the wearer of a circular cloak a trifle discomfited, but if they are made nice and full they have many advantages. The bonnet on this figure is a neat straw bonnet; it is trimmed with a bow of ribbon the colour of the uniform cloak, and has strings of white ribbon. Outdoor uniforms, when worn by nurses who appreciate neatness of person, are as picturesque as they are practical.

### Everybody's Opinion.

### THE NURSES' CO-OPERATION.

MISS MARY M. BELCHER writes: Miss Honnor Morten was one of the members of the Provisional Committee of the Nurses' Co-operation. From the first she took the deepest interest in the scheme, and aided greatly towards its successful completion. It is due partly to her labours as Hon. Secretary that the affairs of the Co-operation have worked so well and so successfully during the first year of its existence. The absence of Miss Morten's name from the annual report is due to the fact that, owing to her well-known literary experience, the drawing up of all papers relating to the society was left almost entirely in her hands. I am glad to be able to express my gratitude for the very ready help and sympathy that mitigated so greatly the anxiety inseparable from any arge work of organisation.

### MALE PROBATIONERS.

"ENTERPRISE" writes: May I ask if any of my fellow nurses can tell me of one or two hospitals which receive male probationers. I take the greatest interest in nursing and am very wishful for an hospital training. I read the male nurse's correspondence last week in THE HOSPITAL, and fully sympathise with all he said. The need will never exist for us to become as large a body as our nursing sisters, but those of us who are nurses would like to be able to call ourselves "thoroughly trained certificated nurses."

### WOULD-BE SUICIDES.

"D" writes: The little tale in this week's HOSPITAL reminded me of two would-be suicides I have known; their cases often make me think that perhaps most suicides regret the deed as soon as it is done. I asked a woman who had jumped from a bridge into the river what she thought as she was falling? "I wondered if the gentleman at the side of the water would be in time to save me; he was, and I was very glad," she answered. A man who threw himself in the river called to those who ran to rescue him "don't trouble, I can get out by myself," and did so. A curious case of suicide occurred in a friend's house. A young servant with no cause for depression, cut her throat; on recovery she was spoken to about the sinfulness of such an act in which she agreed, and was horrified when told she had done it, as she had not the slightest recollection of it, but as soon as she was quite well and about her work she did it again effectually and died. How could such a suicide be accounted for, as she had no consciousness of ever having had such an inclination?

### A WALKING TOUR.

"TRAMP" writes: I wish some more nurses would give their holiday experiences. Personally I think to choose a congenial companion and plan a walking tour quite the most satisfactory way of spending a holiday. I have never done it yet, but it sounds delightful, and even if the weather were unkind it would be very pleasant to put up at a country inn for a few days; but then I am a "town bird." Have any of your readers ever seen a little book called "Field Paths and Green Lanes," by Louis J. Jennings? It was lent to me, and on the strength of it I mean to try my tour in Surrey and Sussex.

### Appointment.

WEST HAM HOSPITAL, STRATFORD, E.—Miss M. T. Ratliff has been appointed Matron at this hospital, and entered on her duties on April 21st. Miss Ratliff trained at the "London," and afterwards did private nursing for some years. She has worked as charge-nurse in the Whatman ward for a year at the West Kent Hospital, and as Sister at the Staffordshire General Infirmary, on the female side of the institution.

### Notes and Queries.

### Query.

*Sister Hilda.*—Can anyone tell me of a home for a helpless incurable child of three years? Can pay £16 to £20 a year. Particulars given.

### Answers.

*Dorothy.*—Nurses' Residential Club, 92, Charlotte Street, Fitzroy Square, or Nurses' Hotel, 18, Royal Avenue, Chelsea, S.W. The first is just opening now; you will get every particular from secretaries.

*J. C.*—In either infirmaries you mention the training is first-class. You could not do better.

*Nurse X.*—The hospital you name appoints eight resident pupils sessionally. It is Unsectarian. There are 297 beds. Yes, it is nursed by sisters of mercy. Personally we do not know about last part of question; write to hospital for rules.

*F. B. C.*—Of course very glad to help you. There are various country hospitals and some London infirmaries, in which the training is excellent, into which very often admittance is gained without as much delay as there is sometimes with the large London hospitals owing to the numerous applications. Are you sure you are strong? Watch our advertisement columns. Do not enter for less than two years' training, for three if you can do so. When you see what you think suitable, write to Matron for particulars. Let us hear again how you succeed.

*Pater.*—Please communicate your name and address to the Editor, and your questions will be answered in the following number.

### Nursing Homes.

### II. ST. THOMAS'S HOSPITAL.

During the whole of the first year of their training all the probationers at this hospital are lodged in the Nightingale Home, where they have separate bedrooms, and take their meals in a dining-hall situated on the ground floor. A capital room it is too, light, airy, and daintily clean; within it is another room where the probationers can sit when off duty, and receive their friends, and here also the classes of instruction in theoretical nursing are held, and a book case, furnished with neatly-covered volumes bearing the names of standard works on Elementary Anatomy, Physiology, Medical Dictionaries, &c., shows us that every branch of a nurse's training receives due attention. During the summer months the pleasant terrace overlooking the river furnishes a most attractive recreation ground. The river Thames, though often much abused, can never be accused of being unpicturesque at this point. When the sun is kindly and throwing his loveliest tints down on the river, the craft of every sort, and the barges with their great brown sails are the life of a picture which many a rich man may envy a Nightingale probationer. The staff nurses' dormitories are situated at the top of some of the blocks of wards; they are rooms of a good size, with fine views from the windows, and they have recently been converted into distinct rooms with pretty-coloured painted walls instead of partially partitioned off cubicles which was the method which had originally obtained favour. On each floor are the usual bath-rooms, and also the unusual comfort of a small sitting-room, where a nurse can work or read more quietly than in the charming new sitting-room which is common to all the staff nurses, and which is conveniently situated on the ground floor and near to the Matron's office. The night nurses are similarly accommodated to the day nurses, but, of course, in different parts of the building.

### III.—WESTMINSTER.

Westminster Hospital Nurses' Home is situated in Queen Anne's Gate, and, therefore, quite away from the hospital, with which it is connected by a telephone; it is as quiet and unpretentious as it is cheerful and comfortable. The neat beds, each in a separate chamber, the bath-rooms on every floor, the pleasant nurses' sitting room facing the street, the dining-room with its long table, and the convenient little apartment where the nurses' boxes are stored, all merit most hearty praise. It is not possible for every hospital, on account of construction and want of room, to provide its nurses with separate bed-rooms, the authorities are always delighted to do anything in the shape of improvement, but really the public must take part of the responsibility in the shape of providing funds. Separate bed-rooms are a luxury and almost a necessity, for while one nurse feels tired and ready for sweet slumber, another may feel equally wide-awake and be ready to converse with her next door neighbour; so cubicles are a difficulty always. We all know the feeling that one cannot possibly wait till the next day to make some important communication to one's "quondam chum" (which would not upset the tenour of one's life if it were never made at all!), and to feel there is only a wooden partition between oneself and the chum—who can resist the temptation? The private nurses at Westminster are accommodated in exactly the same way as the hospital ward nurses, so that they return from their cases to a veritable "home." We have always noticed that when Westminster nurses meet they have invariably something pleasant to say about the kindliness and comfort which pervades this place, and such unanimity of opinion is agreeable to encounter. On descending to the kitchen we find it an ideal apartment, in its own way deserving the admiration which is evidently felt for it by the culinary genius who presides there.

### Among the Institutions.

**Bristol.**—The annual meeting of this town's District Nurses' Society showed a year's good work. The year 1891 commenced with a deficit of £244, which was lessened to the sum of £118 by the kindness of H.R.H. the Duke of Edinburgh, who led the band at a concert given on behalf of the Society, and by other friends. Seventeen districts have been worked by the Society. The Committee have taken No. 2, Berkeley Square, as a residence for the nurses in the near-lying districts, and for the Lady Superintendent.

**Kilmarnock Nursing Association.**—This is a lucky town, for the Nurses' Association turned the year with a balance of £139, after having paid all expenses; this is a novelty, and a very good sort of novelty. District nurses meet with an amount of genuine gratitude, which must make the work a labour of love, and the nurses of this town seem very lucky in this respect. We are glad to see that Kilmarnock folk recognise how much a district nurse can do in the shape of a crusade against unhygienic dwellings.

**The Society of Operators in Massage and Medical Electricity** held their first ordinary general meeting on the 19th inst. Mr. H. Newman Lawrence, the Chairman, opened the proceedings by explaining that the meeting was called in order that the work done by the Council might be made known to the members, and with a view of learning the opinions of the meeting on any points of interest. The first point was the reason of the existence of the Society, and the second their reason for setting up tests of efficiency. Mr. Lawrence prefaced his address by announcing that the Society is now registered, which act he hoped would lend weight and dignity to its standing. The Hon. Secretary kindly sends us the inaugural address, of which the following are extracts: "First then, as to why we exist! No one at all acquainted with the subject can deny that there is a very considerable number of persons of both sexes who either are or profess to be operators in massage and medical electricity. Of this number a portion are trained nurses, and as such have certain advantages which enable them to keep their qualifications prominently before the medical profession, and to some extent before the general public also. . . . The majority of professed operators, however, are not trained nurses, and these, till our Society was formed, had no opportunity of combining for mutual benefit. They were simply a number of units engaged, with more or less regularity, in a work of great importance, but acting entirely without cohesion, with no opportunity of making their qualifications known other than by pestering overworked medical men with visits and letters, and with no protection against ignorant and untrained impostors. Impudence and importunity were the main qualifications for success, while ability, training, and experience were at a discount. (2.) If this Society is to be of any real use, all its members must possess some definite qualification and have passed some standard, which, so far as general qualifications go, shall put us all on an equal footing. This Society has nothing to do with individual teachers or teaching bodies, nor does it desire to become a teaching body itself, but it does consider that for the good of its members and the profession generally it shall require that such operators as desire its full membership shall pass an examination as a test of proficiency, and that those admitted to associateship shall possess a certificate which proves that a fair knowledge of the theory and practice of our art has been obtained. The list of members and associates, which it is intended to publish annually and distribute to medical men and the various hospitals, &c., will, it is hoped, do much to enable medical men to find operators on whom they can rely, and at the same time secure for our members a ready and effective means of bringing their qualifications before those who may require their services."

### Four Months in a Hospital Ward.

### I.—A PERSONAL EXPERIENCE IN A PROVINCIAL HOSPITAL.

Nearly five years ago, broken down physically and financially though still a young man, for twelve months I had battled against the inroads of a wasting disease, until hope had well nigh given way to despair, and weary of the unequal struggle, I had began to long for the end. At this stage it was my good fortune to procure a hospital note, and arrangements having been made for the comfort and well-being of my young wife and child, I braced up my failing courage and presented myself at the outpatients' department of the hospital, and after examination, was passed on to the medical ward therein to be detained as an in-patient. My feelings I cannot describe, for I can only remember that I seemed dazed and unable to realise the position. When I left my loved ones at home, I fixed my mind steadily upon the fact that here all that human skill could do, would be done, and that at the worst, if I must die, I should have the inexpressible comfort of knowing there would be no increase to the already heavy burden of medical indebtedness my long illness had accumulated. To this view I clung from first to last, and it carried me through. It gave me peace of mind, and after the first strangeness had worn off, I felt as I lay in that clean, airy ward, happy, contented, and grateful. I do not know how it may be with other patients similarly situated, but with me the removal of all anxiety on the subject of the further increase of hopeless indebtedness was an inexpressible relief, and I am strangely mistaken if it is not a most valuable aid to the skill and nursing brought to bear upon all hospital cases.

### "Inclined to Run Away."

I ascended a broad stone staircase, and on entering the medical ward to which I had been directed, made my way with an aching heart to a couch near a fire, and sat down. As soon as I dared I looked around me, and there on a bed close at hand lay a young man in the last stage of consumption. He was delirious, his eyes staring horribly—it was a shock to me, and I was sadly inclined to run away from it all there and then; but at that moment a nurse came up to me, and in womanly, sympathetic tones, which I shall never forget, asked me the nature of my illness, and then, pointing out my bed, advised me to go to it. She looked so gentle and spoke so kindly that already the phantoms I had conjured up began to fade, and the first stage of reconciliation was reached. I afterwards learned this was a charge nurse from another ward doing duty in this department during the holiday of the regular charge nurse. By the side of my bed was a pedestal or "locker" containing two divisions, and into this I put my clothes, and was soon comfortably ensconced between deliciously clean linen sheets.

### Returning Confidence.

Now with returning confidence I took a general survey of my surroundings, and found myself an occupant of one of seventeen beds, all covered with scarlet blankets, symmetrically folded at the ends, with a broad band of snow-white linen sheets turned over, neat and trim, and as true in line as a battery of field artillery at "attention," the simile being still further heightened by the scarlet bed-jackets of the patients. By the side of each bed, in addition to the "locker," was a stout Windsor chair, polished like a mirror. It was a fine lofty ward, lighted by six large windows, all on the one side, and as my bed was opposite to them I could at least see the blue sky as I lay; it was all I saw of the outer world for many weeks. The seventeen beds were ranged down each side, leaving a broad aisle between of highly polished oak, as rich in colour as an old mahogany dining table. There were two smaller wards with a total of seven beds on the same corridor, devoted to medical cases, and also six beds in a composite ward, shared with the surgical department; in all about thirty beds available for medical cases, and under the supervision of our charge nurse.

At each end of the ward was a large fire-place with bright fires burning therein night and day. Some of the windows were lowered at the top, giving us an agreeably pure atmosphere with an even temperature. The walls were coloured a pale sea-green above a painted dado, and hung with a number of framed engravings, and a book-shelf filled with well-thumbed volumes. Plants and ferns stood on each window-sill, and in the centre of the ward was a table with inkstand and other requirements, all polished and shining. At one end were two small rooms partitioned off from the ward; one the clinical room, where tests were made, the other a cloak-room for the heavier garments of the patients, and between them a door leading into the lavatory. By one of the fires was a couch and a table upon which the convalescent patients took their meals. Over each bed hung what was called the "bed board," a piece of wood about twelve inches square upon which was fixed a paper, headed with the name of the honorary physician in charge of the case, printed in large letters, and upon which was written the name and address of the patient occupying the bed, the diagnosis, history, and prescribed treatment of the case, with a note of the diet to be given.

### Smooth Sailing.

This, I think, completes the observations I was able to make during the first half hour of my tenancy of a hospital bed, and the general impression they made upon my mind was one of scrupulous cleanliness, order, and quiet, and I already could see that instead of having to "rough it," as is commonly supposed, the sufferer is, in these institutions, in nine cases out of ten, in much smoother sailing than he would be at his own home. I had been in bed about half an hour when the house doctor and charge nurse of the ward came up to me, and the doctor, after making a searching examination in the way doctors usually do, asked me a number of questions, short and to the point, made entries on the "bed board," hung it up over my head, and without a word as to what he thought, walked quietly away. This was a new experience! Was I, then, not to know anything about his view of my ailments? to one whose misfortune it had been to consult many doctors, it seemed strange; but when later on I came to see the astounding assurance of one or two querulous patients who, always behind the doctor's back, insisted they were being wrongly treated, I could well understand how detrimental to our own welfare and to the good order of the ward it would be, to give us the licence of private patients, and, indeed, I question whether "private patients" would not thrive better were doctors more reticent. The doctor having left the ward, I took down the "bed board" and sought for information in that way, but his notes were so full of technicalities, that with the exception of my name and address, it might have been Arabic for all I could make of it.

### A Nauseous Cargo.

Every day at about this time a probationer comes round with a basket and collects those of the medicine bottles that want replenishing, and such of the "bedboards" as have had additions or alterations noted thereon. These she takes down to the dispensary, from whence in due course the basket re-appears laden with its nauseous cargo for distribution. At one o'clock I noticed the entrance of a probationer and ward maid carrying in a bright tin box, and another probationer following with a large can of milk; this meant dinner. They disappeared into the clinical room, and in a few minutes emerged with plates of smoking hot food, which they carefully distributed, referring occasionaly to the "bed-boards" for guidance. Full diet, light meat diet, light diet, and low diet, those were the divisions, and what the doctor specified on the "bed-boards" from time to time had to be adhered to. Now and then the "light diet" would be supplemented by rations of game, this occurring when some good Samaritan among the neighbouring gentry would generously devote a tithe of his sport to the encouragement of the failing appetites of those to whom such delicacies are as valuable as they are beyond reach.

*(To be continued.)*

### A True Story of Flowers.

"Measure thy life by loss instead of gain.

. . . . . . . . . .

For love's strength standeth in love's sacrifice,
And whoso suffers most hath most to give."

" . . . As reward, the prospect of our future grows into infinite glory, the thought of human nature rises into an elevation unconceived; God appears before us infinite afresh in tenderness; and the darkness of human sorrow, all the sad failure and agony of life, shining with the brightness of Christ's own sacrifice, are changed into the instruments and prophecies of joy!"—Hinton's "Mystery of Pain."

The life of a Royal Princess was fading slowly away. The angels brought the message in the winter time; when the rime was on the trees, and the woods were like fairyland, and the pure snow lay as a cover for the sins of the world. The Royal Princess had always led an active life, and she longed to work in the battlefield, and help others stem the awful tide of sorrow, and sin, and shame. She thought this was following the Father's Will in the most perfect way. Had not the order gone forth? "Go, work in the harvest field of the world."

Was she not a King's daughter? Did she not belong to a Royal Sisterhood? Was it not the wish of her life to help others along the King's highway? It mattered not to the King whether she was rich, or poor; of a high, or low estate. She was his daughter all the while. But the work was hard, and oftentimes she sat down by the wayside weary; there were so many marks of failure along the path. Briars and thorns beset her path, yet—she was working for others, and that was all she cared for.

Then a hard order came—the hardest of all—straight from the White Throne.

"Stand still," whispered the angel. "Stand still and suffer, for the Master loves you."

It is the order that "comes straightest from God's hand and makes us feel Him nearest to ourselves."

The Princess hardly noticed, for the whisper came by such slow degrees, only each day there was a little more "giving up," a little more work left undone. The song of life was set in a minor key without her heeding the change.

Then the weary fight began, and she went even through the Valley of the Shadow of Death, to the very borders of the kingdom which is beyond the sky. She "thought she heard the King's voice." On the border—just as she reached the Land of Lilies—she was called back by a tone which was stronger than death.

The Princess sighed . . . but she was very glad. Only that was a long, long time afterwards. Meanwhile, she "set her soul to suffer perfectly." It was as if she was resting in the Father's arms, and the flowers helped her. Flowers were the talisman which gave her courage and faith. Flowers whispered of hope along that path of pain and suffering. Flowers told a story of the unceasing love of the Father. She knew they were the King's jewels, so she watched them by her bedside as she lay still and silent there.

"Waiting to die," the watchers said, with tears in their voices and an aching at their hearts.

"Hope!" whispered the flowers, lifting their sweet heads so the Princess could not trouble.

"Is it not God's own finger tips
Laid on thee in a tender steadfastness?"

Yes, the Princess was satisfied to trust.

"Spring is coming," said the first snowdrops, hanging their heads modestly. These "gentle heralds" were shy at being the first flowers to show themselves; the snow had sunk into their hearts and purified them, and they bravely gave to winter the "call of death." Trembling they sang out their message, and the Princess listened with a smile on her face. "A King's daughter must be as humble as a snowdrop," she was thinking.

"The sun is shining." It was a little early primrose who spoke, and a throstle answered from a tree outside, "I hear you, I hear you, I hear you." The "wild little poet" had heard someone was ill, so made up his mind to sing his best.

Now the primroses had come the Princess knew the hedges were budding, and she could picture the lambs tails, and palm, and spider webs sparkling with diamonds. "Shall I be up when the bells come?" she wondered to herself.

"The dry leaves
Are tipped by the grass, and so I know
That nature from her delicate ear hath caught
The dropping of the velvet foot of spring."

She could tell by the flowers the weeks were passing, yet the pale Angel of Death stood there. "Ah, well," sighed the breeze, and the throstle in the tree moped.

Just then a bowl of cowslips was placed on the table by the bed, a glory of gold, whose brightness made the primroses fade and die. They in their turn drooped their heads before the gold of the Lent lilies,

"A host of golden daffodils,"

singing the message softly to one another, "The shadows will pass away, and the light of the resurrection will break across the sky. Look up, it is the resurrection life of spring."

"Look up, look up, look up," echoed the throstle.

"After April, when May follows,
And the white-throat builds, and all the swallows!
Hark, where my blossomed pear tree in the hedge
Leans to the field and scatters on the clover
Blossoms and dewdrops—at the bent spray's edge—
That's the wise thrush he sings each song thrice over,
Lest you should think he never could recapture
The first wild, careless rapture!"

It was the time of lilies of the valley, flowers the Princess loved the best. It was then that she would have chosen to die, if the choice had been hers. Lilies would have rested on her grave, and made it white. Everyone sent lilies. They came ringing their tiny peals of bells from north, and south, and east, and west, bringing messages of love and comfort. Sweet fairy bells! She knew they were the bells which the child angels ring in heaven. All love, love, love, never tired of ringing, wafting the holy story of their perfect purity—souls untouched by this world's sins.

One eventide, when the day had been long and dreary, and some of the flowers were tired of blooming, the Princess lay, feeling that Hope was tired too.

"Why we suffer—that is hid
With God's foreknowledge in the clouds of heaven."

She wondered why she had been chosen to suffer, and for a moment she wondered if the King had forgotten her. Yes, forgotten her! As if for reproach the door opened, and someone entered, bearing in each hand an emblem of white. In one hand a bunch of fresh lilies, in the other white lilac—gentle, soft white lilac—which shrinks from the slightest touch, and cannot brave a rough world. They were placed on her bed, and she smiled. Sweet peace came again as the flowers whispered, "He is here." She could not sleep, but "He Himself watches with those who wake," so she rested through the long hours of night.

Her room was as a summer bower, for everyone sent offerings of flowers. Everyone was good to the Princess. There was an auricula growing which a loving heart had reared, and which she watched from day to day.

"Little flower; but if I could understand
What you are, root and all, and all in all,
I should know what God and man is."

She thought to herself; and she realised that at last she could understand, for the flowers were teaching her through suffering.

(To be continued.)

## Figures and Tables

**Figure f1:**
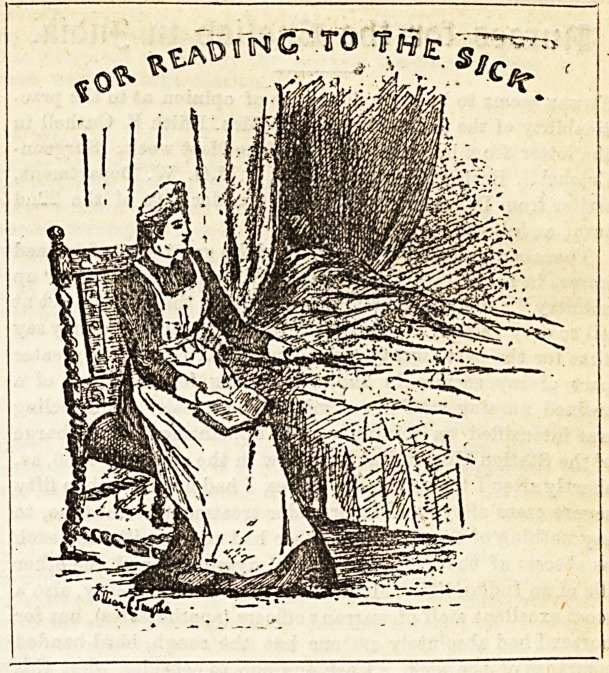


**Figure f2:**